# Capsaicinoids improve consequences of physical activity

**DOI:** 10.1016/j.toxrep.2018.05.001

**Published:** 2018-05-15

**Authors:** Kazim Sahin, Cemal Orhan, Mehmet Tuzcu, Nurhan Sahin, Fusun Erten, Vijaya Juturu

**Affiliations:** a Department of Animal Nutrition, Faculty of Veterinary Science, Firat University, Elazig, Turkey; b Division of Biology, Faculty of Science, Firat University, Elazig, Turkey; c Research and Development, Clinical Affairs, OmniActive Health Technologies Inc., Morristown, NJ, USA

**Keywords:** ACLY, ATP-citrate lyase, ACS, acetyl-CoA synthetase, AMPK, phosphorylated AMP-activated protein kinase, ARE, antioxidant response element, CAPs, capsaicinoids, FAS, fatty acid synthase, GSH-Px, glutathione peroxidase, HO-1, heme-oxygenase 1, IL-10, interleukin-10, LXR-s, liver X receptor-s, MMP-9, matrix metalloproteinase-9, MDA, malondialdehyde, NF-κB, nuclear factor kappa-light-chain-enhancer of activated B cells, Nrf2, nuclear factor (erythroid-derived 2)-like 2, PGC-la, peroxisomal proliferator activator receptor c coactivator, PPAR-γ, peroxisome proliferator-activated receptor gamma, ROS, reactive oxygen species, SOD, superoxide dismutase, SREBP-1c, sterol regulatory element-binding protein1c, TC, total serum cholesterol, Tfam, mitochondrial transcription factor A, TG, triglyceride, TRPV1, transient receptor potential vanilloid subtype 1, TNF-α, tumor necrosis factor-α, Exercise, Capsaicinoid, PPAR-γ, Nrf2, SREBP-1c

## Abstract

The purpose of this study was to investigate the effects of capsaicinoids (CAPs) on lipid metabolism, inflammation, antioxidant status and the changes in gene products involved in these metabolic functions in exercised rats. A total of 28 male Wistar albino rats were randomly divided into four groups (n = 7) (i) No exercise and no CAPs, (ii) No exercise + CAPs (iii) Regular exercise, (iv) Regular exercise + CAPs. Rats were administered as 0.2 mg capsaicinoids from 10 mg/kg BW/day Capsimax^®^ daily for 8 weeks. A significant decrease in lactate and malondialdehyde (MDA) levels and increase in activities of antioxidant enzymes were observed in the combination of regular exercise and CAPs group (*P* < 0.0001). Regular exercise + CAPs treated rats had greater nuclear factor-E2-related factor-2 (Nrf2) and heme oxygenase-1 (HO-1) levels in muscle than regular exercise and no exercise rats (*P* < 0.001). Nevertheless, regular exercise + CAPs treated had lower nuclear factor kappa B (NF-κB) and IL-10 levels in muscle than regular exercise and control rats (*P* < 0.001). Muscle sterol regulatory element-binding protein 1c (SREBP-1c), liver X receptors (LXR), ATP citrate lyase (ACLY) and fatty acid synthase (FAS) levels in the regular exercise + CAPs group were lower than all groups (*P* < 0.05). However, muscle PPAR-γ level was higher in the regular exercise and CAPs alone than the no exercise rats. These results suggest CAPs with regular exercise may enhance lipid metabolism by regulation of gene products involved in lipid and antioxidant metabolism including SREBP-1c, PPAR-γ, and Nrf2 pathways in rats.

## Highlights

• Capsaicinoids (CAPs) are active compounds in *Capsicum* fruits.
• CAPs have anti-inflammatory and antioxidant properties.
• CAPs with regular exercise may enhance lipid metabolism.
• CAPs down-regulate muscle SREBP-1c, LXRs, ACLY, FAS in exercised rats.

## Graphical abstract

**Figure:**
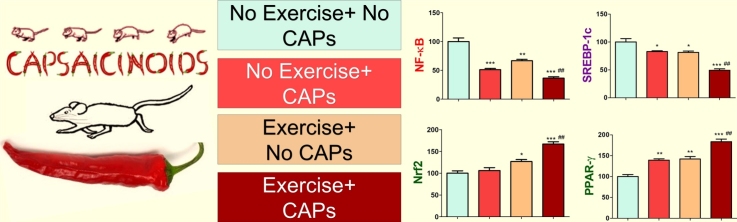

## 1 Introduction

Regular physical exercise has many useful benefits on human health such as prevention of chronic diseases. In recent years, the frequency of chronic diseases has been increasing in the world including disorders of carbohydrate and lipid metabolism [1]. There are many studies showing that regular exercise has positive effects on lipid metabolism as well [[1], [2], [3]]. These studies have shown that regular exercise improves insulin sensitivity, decreases risk factors for metabolic syndrome [4], improves beneficial factors related to muscle metabolism [5], increases brain volume and improves cognitive function [6] and reduces oxidative stress [7,8]. Additionally, physical exercise improves muscle mass and muscle function by increasing muscle protein synthesis and a stimulation of mitochondrial biogenesis [9,10]. However, in some studies conducted in humans and animals exposed to treadmill running at 20 m/min on a 15% incline for 60 min/day (5 days/wk) showed that high-intensity exercise causes oxidative damage to biomolecules, and this process is related to the increased metabolism that occurs when the body is exposed to intense exercise [[11], [12], [13]]. In addition, Spanidis et al., [13] reported that static oxidation-reduction potential marker and total antioxidant capacity was improved in athletes, whereas, thiobarbituric acid reactive substances (TBARS), protein carbonyls and the total antioxidant capacity (TAC) showed obvious variations in the athletes, suggesting that the optimum approach with which to counteract including antioxidant supplementation.

In recent years, carbohydrate and lipid metabolism disorders resulting from inadequate exercise or unbalanced nutrition could induce hyperlipidemia, diabetes and other lipid metabolism disorders by regulating signal pathways including transcription factors [the sterol regulatory element-binding protein 1 (SREBP-1) and liver X receptors (LXR) and several enzymes including ATP-citrate lyase (ACL), acetyl-CoA carboxylase (ACC) and fatty acid synthase (FAS)], antioxidant metabolism such as nuclear factor-E2-related factor-2 (Nrf2) and inflammation e.g. nuclear factor kappa B (NF-κB) [2,14]. Nrf2 is the key regulator of antioxidant signaling, which plays an important role in the regulation of blood sugar and insulin sensitivity [15]. SREBP-1 may control the ectopic accumulation of fat and may activate the target gene FAS, an important enzyme that regulates the levels of fatty acid synthesis [14]. LXRs regulate fatty acid and cholesterol homeostasis and are expressed mainly in the liver and other tissues involved in lipid metabolism [16]. AMP-activated kinase (AMPK), a key regulator of energy metabolic homeostasis, can play an important role in regulating the synthesis of fatty acids, cholesterol, glucose, and hepatic gluconeogenesis [17].

Currently, the use of naturally occurring antioxidants such as coenzyme Q10 [18,19], curcumin [8], quercetin [20] and resveratrol [21] improve performance and exercise capacity during physical exercises. Similarly, polyphenolic extracts with antioxidant properties are also effective to improve antioxidant capacity in endothelial and muscle cells [22]. These antioxidants have a bioenergetics and antioxidant system reinforcement role, induce skeletal muscle and brain mitochondrial biogenesis. Meanwhile, capsaicinoids (CAPs), which have high anti-inflammatory and antioxidant properties and are widely used in traditional medicine, but which are lacking in research on sports supplements. Capsaicinoids are the major pungent, naturally occurring active compounds in *Capsicum* fruits such as hot chili peppers (genus *Capsicum*), with the most abundant forms being capsaicin, dihydrocapsaicin, and nordihydrocapsaicin [23,24]. Capsaicinoids have many biological and physiological activities including anti-inflammatory [25], antioxidant [26], anticancer [27], thermogenesis [28] and lipolysis enhancer [24], hormone function improver [29] and blood glucose regulation [30]. It has also been reported that consumption of food containing capsaicinoids is associated with a lower incidence of lipid metabolism disorders [31].

There are also thermogenic and appetitive effects of capsaicin [32,33]. Janssens et al. [33] reported that consumption of 2.56 mg capsaicin per meal promoted fat oxidation in negative energy balance and did not increase blood pressure significantly. In addition, it was reported that capsiate activates transient receptor potential vanilloid subtype 1 (TRPV1) receptors in the gut but not in the oral cavity [32]. Although the effects of CAPs have been demonstrated in vitro and in vivo studies, the effects of CAPs on the major transcriptional regulator of fatty acid (SREBP-1) and some key transcription factors (NF-κB and Nrf2) have not been fully investigated in exercise-treated rats. Therefore, this study was designed to investigate the effects of exercise and CAPs on lipid metabolism, inflammation and oxidative stress in regularly trained rats. Furthermore, its mechanisms of attenuation of metabolic profiles, inflammation, and oxidative stress were elucidated by evaluating NF-κB and Nrf2 pathways as well as SREBP-1, LXR, ACL, ACC, FAS, and PPAR-γ.

## 2 Materials and methods

### 2.1 Animals and feeding protocols

A total of 28 male Wistar albino rats (n = 7, age: 8 weeks, weight:180 ± 20 g) housed in a controlled standard laboratory environment (12:12-h light-dark cycle at 22 °C) and animals fed with rat chow and water ad libitum. All animal procedures were approved by the Animal Experimentation Ethics Committee of Firat University (Elazig, Turkey). Table 1 provides a composition of the basal (control) diet. Rats were randomly divided into four groups: (i) sedentary control (no exercise and no CAPs), (ii) sedentary control + CAPs [no exercise + CAPs (0.2 mg capsaicinoids from 10 mg/kg BW/day Capsimax^®^), (iii) exercise, (iv) exercise + CAPs. CAPs administered as 0.2 mg capsaicinoids from 10 mg/kg BW/day Capsimax^®^ daily for 8 weeks. Capsimax consists of capsaicinoids (CAPs) obtained from dried red fruits of Capsicum annuum L. The product (Capsimax) contains standardized 2% capsaicinoids (wt/wt), of which 1.2–1.35% is capsaicin, 0.6-0.8% is dihydrocapsaicin, and 0.1-0.2% is nordihydrocapsaicin. Capsaicinoids concentrate (Item code: 3822; Batch No: CFEB-07070003) was provided by OmniActive Health Technologies, Ltd., India. The dosage was chosen based on previously reported dosage in rodents [34].

**Table 1:** Composition of basal diet.

Description	%
Cracked Barley	30.2
Soybean meal	10.0
Sunflower meal	38.0
Wheat bran	6.0
Molasses	10.0
Limestone	3.0
Salt	1.5
DL-Methionine	0.8
Dicalcium phosphate	0.3
Vitamin and mineral premix^a^	0.2
Analysis (dry matter bases)	
ME, kcal/kg	3.088
Crude protein, %	24.3
Ether extract, %	3.4
Crude fiber, %	6.9
Ash, %	8.1
Ca, %	1.3
P, %	0.9

a The vitamin-mineral premix provides the following (per kg): all-trans-retinyl acetate, 1.8 mg; cholecalciferol, 0.025 mg; all-rac-a-tocopherol acetate, 125 mg; menadione (menadione sodium bisulfate), 1.1 mg; riboflavin, 4.4 mg; thiamine (thiamine mononitrate), 1.1 mg; vitamin B-6, 2.2 mg; niacin, 35 mg; Ca-pantothenate, 10 mg; vitamin B-12, 0.02 mg; folic acid, 0.55 mg; d-biotin, 0.1 mg. manganese (from manganese oxide), 40 mg; iron (from iron sulfate), 12.5 mg; zinc (from zinc oxide), 25 mg; copper (from copper sulfate), 3.5 mg; iodine (from potassium iodide), 0.3 mg; selenium (from sodium selenite), 0.15 mg; choline chloride, 175 mg.

### 2.2 Exercise protocol

The exercise protocols performed on a motor-driven rodent treadmill (MAY-TME, Commat Limited, Ankara, Turkey). The treadmill included a stimulus grid at the back end of the treadmill which provided an electric shock if the animal placed its paw on the grid. The apparatus consisted of a 5-lane animal exerciser utilizing single belt construction with dividing walls suspended over the tread surface. All exercise tests were performed during the same time period of the day to minimize diurnal effects. All rats were pre-trained in order for the animals to be exposed to the treadmill equipment and handling for 1 week. The exercise period based on (i) 1st day, 10 m/min, 10 min, (ii) 2nd day, 20 m/min, 10 min, (iii) 3rd day, 25 m/min, 10 min, (iv) 4th day, 25 m/min, 20 min and (v) 5th day, 25 m/min, 30 min. After 1-week treadmill familiarization to eliminate novel and stress effects, animals in treadmill exercise groups ran on the treadmill 25 m/min, 45 min/day and five days per week for 8 weeks according to the protocol published earlier [8]. None of the rats were subjected to harm during the endurance tests in this study.

### 2.3 Sample collection

At the end of the experiment, all rats were subjected to overnight fasting and blood and slow-twitch muscles (soleus and gastrocnemius deep portion) specimens were taken from decapitated animals via cervical dislocation via anesthesia. This procedure was carried out 48 h after the last exercise session to avoid the metabolic effects of the final run. Blood samples were collected by gel biochemical tubes and serum samples were taken and centrifuged at 4 °C at 2370 x g for 10 min in a chilled centrifuge. In addition, the tissues obtained from the animals were stored in a deep freeze at −80 °C until analysis.

### 2.4 Laboratory analyses

Serum glucose, aspartate aminotransferase (AST), alanine aminotransferase (ALT), urea, creatinine, total cholesterol and triglyceride levels were analyzed with a portable automated chemistry analyzer (Samsung LABGEO PT10, Samsung Electronics Co., Suwon, Korea). Reproducibility and device/method sensitivity of LABGEOPT10 were established according to IVR-PT06 guidelines. The concentration of serum lactate was measured with the rat Lactate Assay Kit (Cayman Chemical Co., Ann Arbor, MI, USA) by ELISA (Elx-800, Bio-Tek Instruments Inc, Vermont, USA). The interassay and intraassay coefficients of variation were 5.1% and 8.6% for lactate.

Muscle MDA concentrations were measured according to the previously described method [8] with a Shimadzu UV–vis SPD-10 AVP detector, a CTO-10 AS VP column and 30 mM KH_2_PO_4_ and methanol (82.5: 17.5, v/v, pH 3.6) at a flow rate of 1.2 ml/min. Column waste was monitored at 250 nm. Tissue homogenization (10%, w/v) in 10 mM phosphate buffer (pH 7.4) was prepared and centrifuged at 14,000 x g for 10 min at 4 °C. The resulting supernatant was collected and kept at -80 °C for MDA estimation.

Activity of total SOD in the muscle tissue [in 20 mM HEPES (N-2 hydroxy-ethyl piperazine-N'-2-ethanesulfonic acid) buffer, 1 mM ethylene glycol tetraacetic acid, 210 mM mannitol, 70 mM sucrose, pH 7.2 per g of tissue] was determined by a commercial kit (Cayman Chemical, Ann Arbor, MI, USA) according to the manufacturer's instructions. The supernatant was collected after centrifugation at 12.000 *g* for 20 min at 4 °C. The supernatant was purified from the salt by passing through a Sephadex G-25 column. The samples were also treated with a mixture of ethanol-chloroform (2: 1, v/v) and distilled water to remove hemoglobin and red blood cells and the absorbance plate was read on a reader (Biotek Instruments, Inc. Vermont, USA) at 450 nm. The results were expressed as units per mg protein (U/mg protein) using standard calibration curve. The activity of GSH-Px was analyzed according to the manufacturer's instructions (Cayman Chemical, Ann Arbor, MI, USA). Muscle tissue was homogenized with the polytron homogenizer in cold buffer (50 mM Tris-HCl, pH 7.5, 5 mM EDTA and 1 mM dithiothreitol) per tissue and then subjected to centrifugation at 10,000 *g* for 15 min at 4 °C. This method is based on the oxidation of NADPH to NADP^+^, which is accompanied by an absorbance drop at 340 nm for 5 min GSH-Px activity was measured by initiating the reaction with 2.4 mM cumene hydroperoxide. One unit is defined as the amount of enzyme that oxidizes 1 μmol of NADPH per min at 25 °C. The absorbance was read every minute at 340 nm using a plate reader (Biotek Instruments, Inc. Vermont, USA) to obtain at least 5-time points. The GSH-Px activity was calculated in nmol/min/mg of protein using standard calibration curve.

### 2.5 Western blot analyses

Protein extraction was performed by standardizing the muscle in 1 ml of ice-cold hypotonic buffer (buffer A) containing 10 mM HEPES, 1 mM dithiothreitol (DTT), 0.1 mM EDTA and 0.1 mM phenylmethylsulfonyl fluoride (PMSF) for Western blot analysis. The homogenate was mixed with 80 μl of 10% Nonidet P-40 (NP-40) solution and then centrifuged at 14,000 × g for 2 min. The precipitates were washed once with 500 μL Buffer A and 40 μL 10% NP-40, centrifuged and resuspended in a 200 μL buffer containing 50 mM HEPES at pH 7.8, 50 mM KCl, 300 mM NaCl, 0.1 mM EDTA, 1 mM DTT, 0.1 mM PMSF, and 20% glycerol) and re-centrifuged at 14,800 × g for 5 min. The supernatant was collected and used for the determination of NF-κB, Nrf-2, HO-1, total AMPK, p-AMPK, PPAR-γ, SREBP-1c, LXRs, ACLY and FAS according to the previously described method [8]. Briefly, 50 μg of proteins were electrophoresed and then transferred to a nitrocellulose membrane (Schleicher and Schuell Inc., Keene, NH, USA). The phosphorylated form of antibodies against NF-κB, Nrf-2, HO-1, total AMPK, pAMPK, PPAR-γ, SREBP-1c, LXRs, ACLY, FAS (Abcam, Cambridge, UK) were diluted (1:1000) in the same buffer containing 0.05% Tween-20. Protein loading was controlled using monoclonal mouse antibody against β-actin (A5316; Sigma). The bands were examined densitometrically using Image J, an image analysis system (National Institute of Health, Bethesda, USA).

### 2.6 Statistical analysis

Data were stated as mean ± SE. The alteration among groups was analyzed using one-way analysis of variance (ANOVA) followed by the Tukey *post hoc* test (SAS Institute: SAS User’s Guide: Statistics), and P < 0.05 was considered statistically significant.

## 3 Results

### 3.1 Exhaustion time and biochemical parameters

**Fig. 1:**
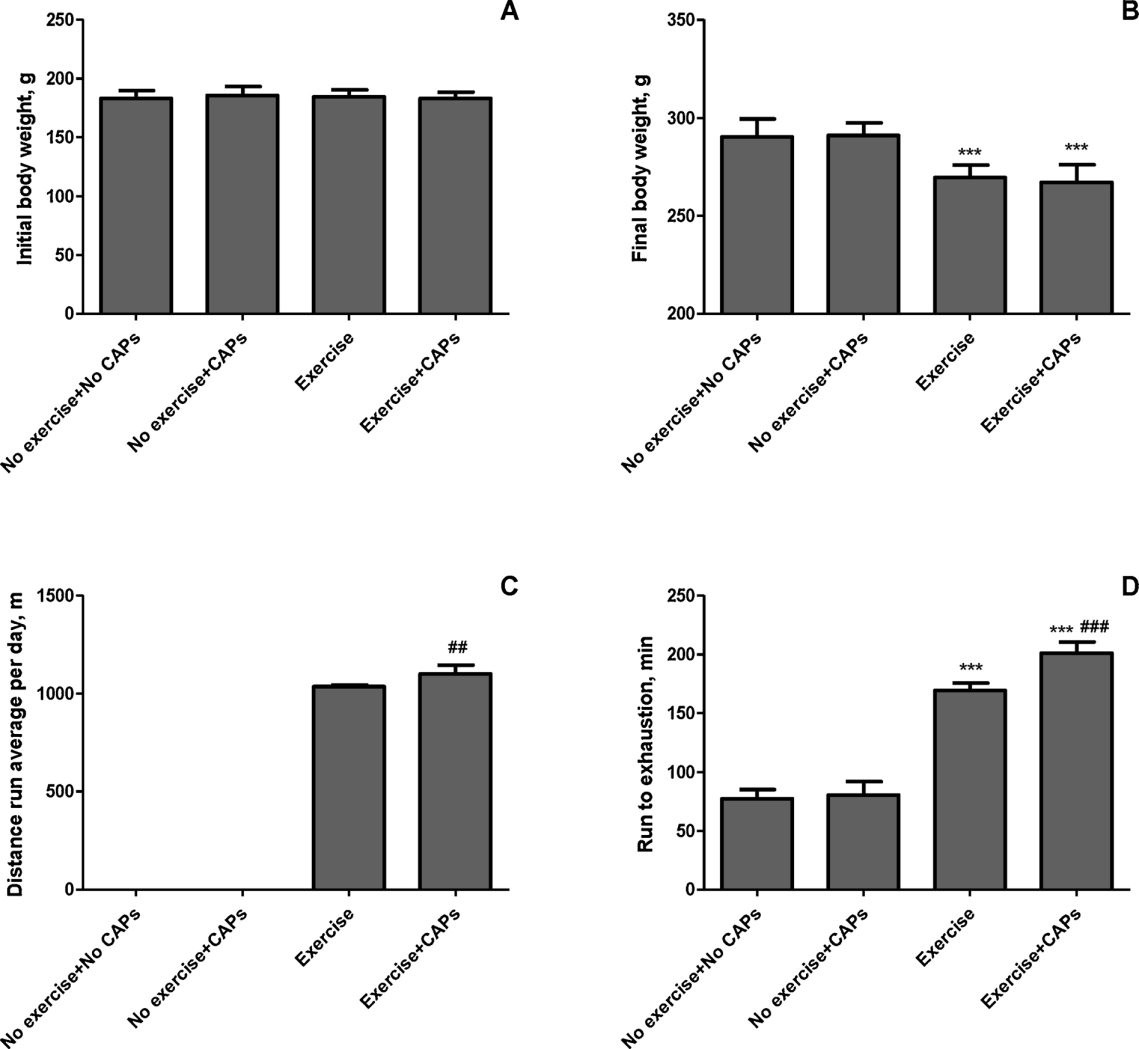
Effects of Capsaicinoids (CAPs) supplementation and regular exercise on body weight (initial BW Panel A and final BW Panel B), distance run average (Panel C) and exhaustion time (Panel D) in rats. Each bar represents the mean (n = 7) and standard error. *** p < 0.001 as compared with control group; ## p < 0.01, ### p < 0.001 as compared with exercise group.

**Fig. 2:**
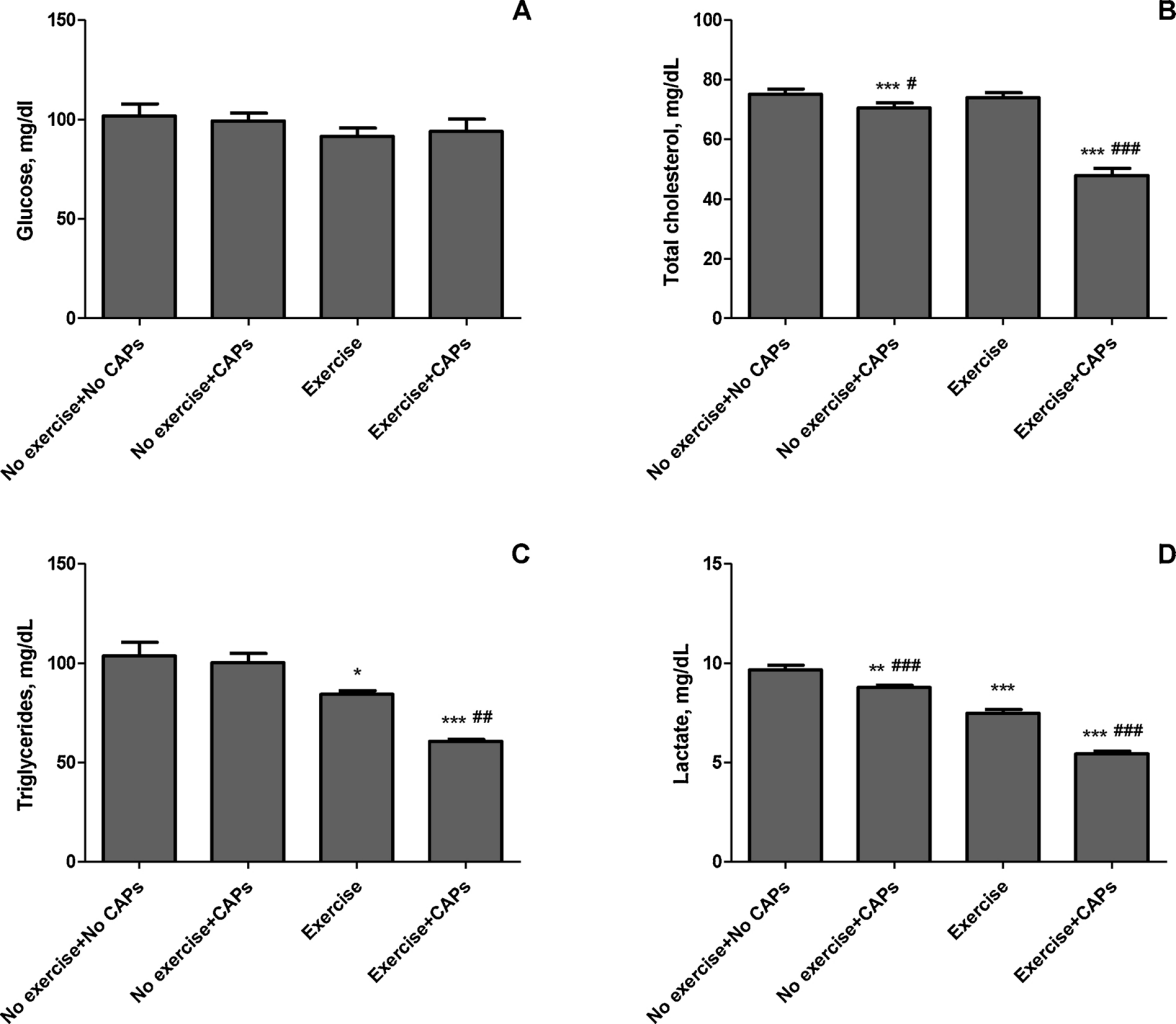
The effects of Capsaicinoids (CAPs) on serum biochemical parameters in regular exercise-trained rats. Panels A–D show the level of blood glucose (Panel A), total serum cholesterol (Panel B), triglycerides (Panel C), and lactate (Panel D) in various groups. Each bar represents the mean (n = 7) and standard error. * p < 0.05, ** p < 0.01, *** p < 0.001 as compared with control group; # p < 0.05, ## p < 0.01, ### p < 0.001 as compared with exercise group.

As seen in Fig. 1, a significant difference in final body weight and time to exhaustion between the control (no exercise and no CAPs) and exercise rats was observed (*P* < 0.001). Final body weight decreased in exercise and combination of regular exercise and CAPs rats (*P* < 0.01). However, CAPs alone (no exercise + CAPs) did not affect body weight (*P* > 0.05). Capsaicinoids supplementation increased the time to exhaustion in the regularly exercised rats (18.8%; *P* < 0.05). CAPs only rats showed exhaustion times similar to that of the control group of rats. No significant differences in safety end markers for liver and kidney function tests in all treatments (*P* > 0.05). Fig. 2 shows metabolic health parameters including glucose and lipid profile in all treatments. No significant difference in blood glucose concentrations was observed between all groups (*P* > 0.05). Additionally, serum total cholesterol decreased in CAPs treated group significantly compared with other groups (*P* < 0.05). Exercise + CAPs group reduced total cholesterol much more than no exercise + CAPs group (*P* < 0.05). CAPs alone did not decrease triglyceride compared with the control group (*P* > 0.05). However, regular exercised rats had less serum triglyceride (103.71 vs 84.29 mg/dL; 18.7% *P*<0.0001) concentration than control rats. Serum lactate levels in regular exercise group much lower than those of control and no exercise + CAPs treated rats (9.66 vs 7.47; 22.7%, *P* < 0.001). Serum lactate levels were also lower in the Exercise + CAPs group than in the controls and exercise groups (43.8% and 27.3%; *P* <0.0001, Fig. 2).

### 3.2 Muscle MDA and antioxidant enzymes

**Fig. 3:**
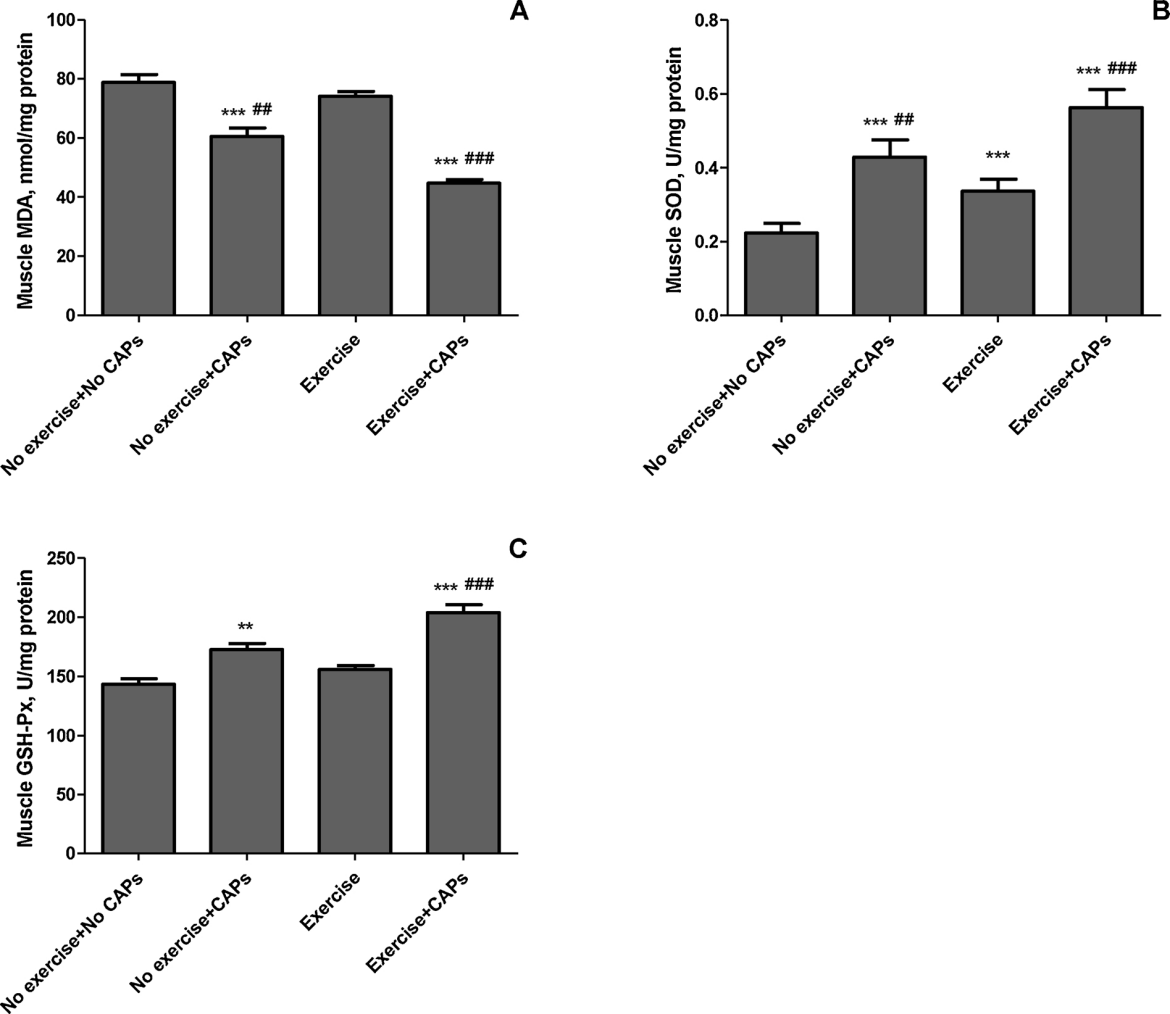
The effects of Capsaicinoids (CAPs) on muscle lipid peroxidation [Panel A: muscle malondialdehyde (MDA)] and antioxidant enzymes [Panel B: muscle three forms of superoxide dismutase (SOD) (mitochondrial, cytoplasmic, and extracellular); Panel C: Glutathione peroxidase (GSH-Px)] in regular exercise-trained rats. Each bar represents the mean (n = 7) and standard error. ** p < 0.01, *** p < 0.001 as compared with control group; ## p < 0.01, ### p < 0.001 as compared with exercise group.

Muscle malondialdehyde (MDA) concentration decreased by 21.8% (*P* < 0.0001; Fig. 3) in CAPs group compared to control rats. Exercise + CAPs group treatment reduced by 42.3% and 39.2% in muscle MDA concentration compared to control and regularly exercised rats (*P* < 0.0001). Regular exercise rats had higher muscle total superoxide dismutase (SOD; 34.1 vs 22.7; 50.1% *P* < 0.0001) activity (U/mg protein) than control rats. However, regular exercise rats did not have significant higher GSH-Px than that of the control group (*P* > 0.05). Decrease in MDA and increase in muscle SOD and GHS-Px concentrations in response to regular exercise + CAPs treatment was more notable than other groups (*P* < 0.001 for all; Fig. 3).

### 3.3 Muscle protein levels

**Fig. 4:**
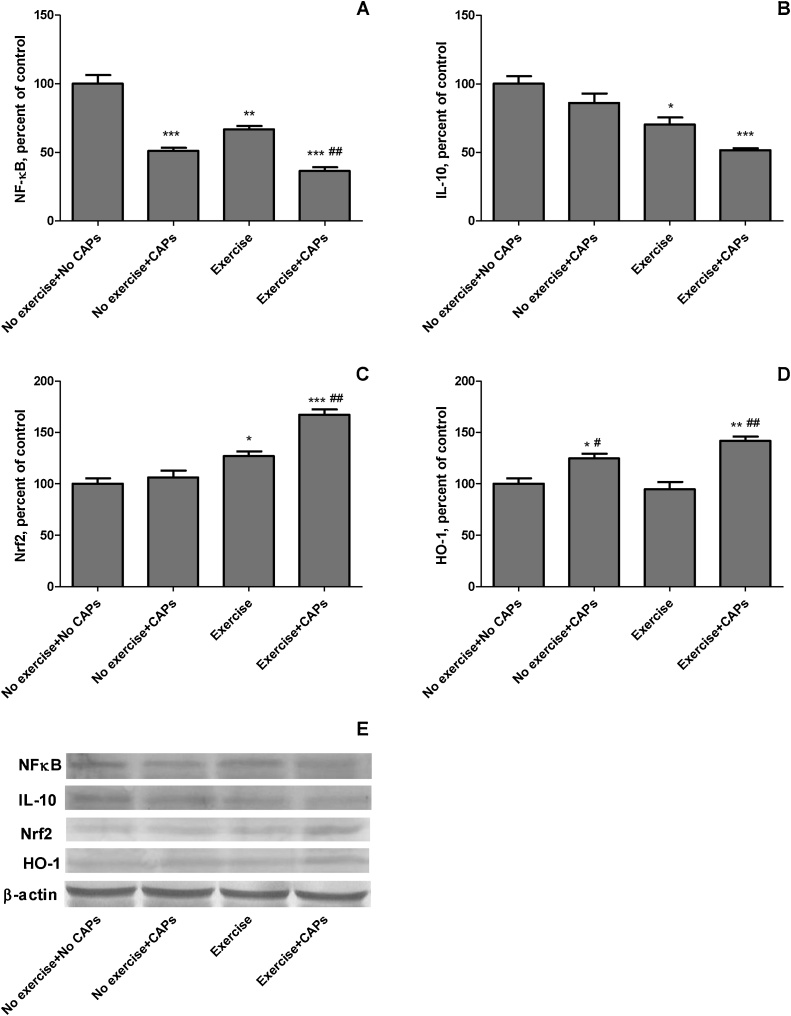
The effects of Capsaicinoids (CAPs) on muscle protein levels of anti-inflammatory and antioxidant markers of NF-κB (Panel A), IL-10 (Panel B), Nrf2 (Panel C), and HO-1 (Panel D) in exercise-trained rats determined by immunoblot analysis. The intensity of the bands shown in Panel E was quantiﬁed by densitometric analysis. Data are expressed as a ratio of normal control value (set to 100%). Each bar represents the mean and standard error. Blots were repeated at least 3 times (n = 3) and only a representative blot is shown in Panel E. β-Actin was included to ensure equal protein loading. * p < 0.05, ** p < 0.01, *** p < 0.001 as compared with control group; # p < 0.05, ## p < 0.01, ### p < 0.001 as compared with exercise group.

Regular exercised rats had less the level of NF-κB and IL-10 in gastrocnemius muscle than control rats (Fig. 4- Panel A and B; *P* < 0.05). Regularly exercised rats had greater Nrf2 levels in muscle than control rats (*P* < 0.001; Fig. 4 Panel C). However, there were no significant differences in HO-1 levels between regular exercised rats and control rats (Fig. 4 Panel D; *P* >0.05). There was 27.0% increase in Nrf2 levels of muscle in response to exercise and there was 33.4% and 29.7% a decrease in NF-κB and IL-10 of the muscle of in exercised rats compared with the control group. Regular exercise + CAPs treated rats had greater Nrf2 and HO-1 levels in muscle than regular exercise and control rats (*P* < 0.001). Nevertheless, regular exercise + CAPs treated had lower NF-κB and IL-10 levels in muscle than regular exercise treated and control rats (*P* < 0.001). There were mean increases in levels of muscle Nrf2 and HO-1 by 31.6% and 49.4%, respectively and decreases in muscle NF-κB and IL-10 levels by 45.3% and 26.7% in regular exercise + CAPs treated rats when compared with regular exercise (*P* < 0.001 for all).

**Fig. 5:**
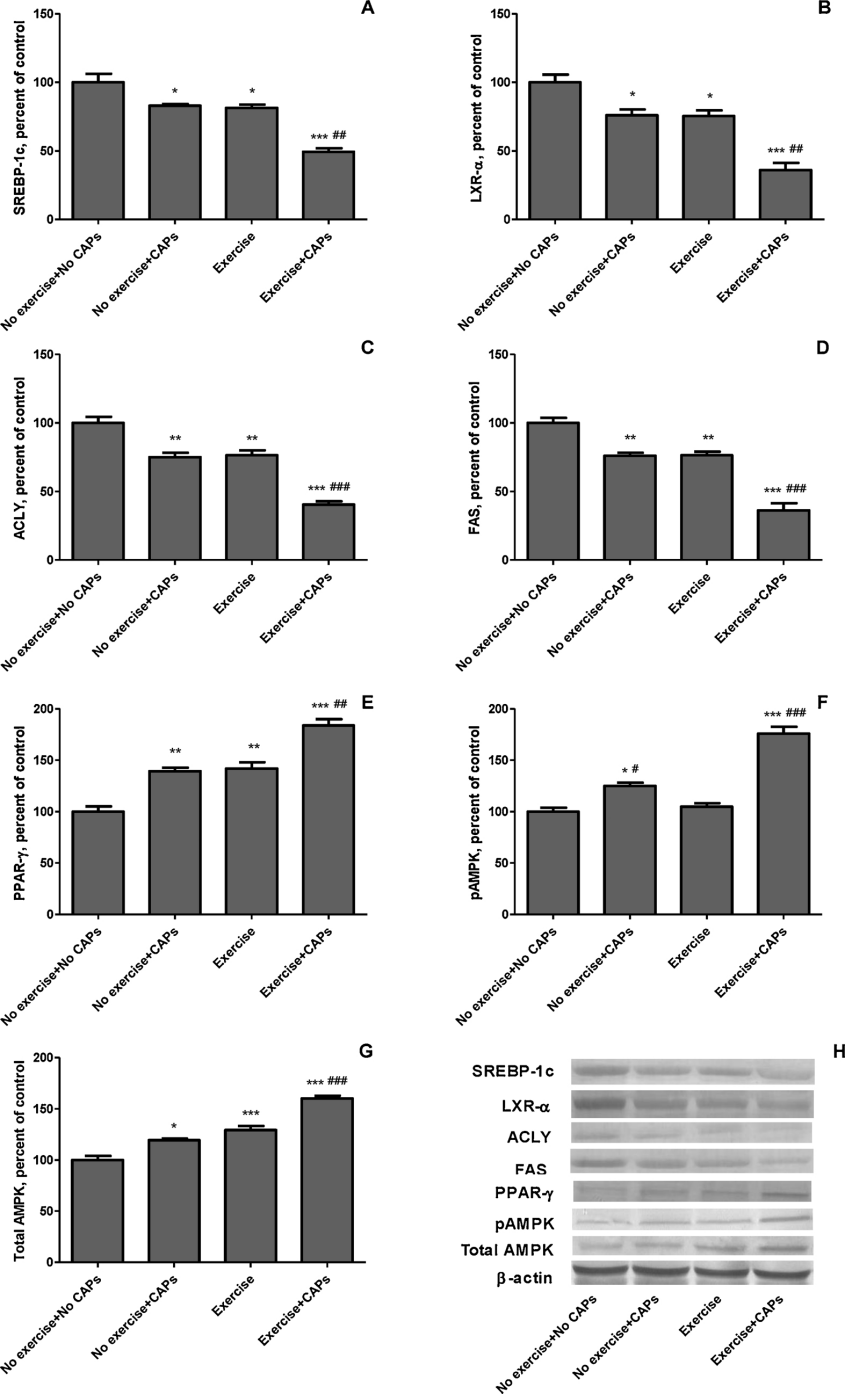
The effects of Capsaicinoids (CAPs) on muscle protein levels of SREBP-1c (Panel A), LXRs (Panel B), ACLY (Panel C), FAS (Panel D), PPAR-γ (Panel E), pAMPK (Panel F), and total AMPK (Panel G) in exercise-trained rats determined by immunoblot analysis. The intensity of the bands shown in Panel H was quantiﬁed by densitometric analysis. Data are expressed as a ratio of normal control value (set to 100%). Each bar represents the mean and standard error. Blots were repeated at least 3 times (n = 3) and only a representative blot is shown in Panel H. β-Actin was included to ensure equal protein loading. * p < 0.05, ** p < 0.01, *** p < 0.001 as compared with control group; # p < 0.05, ## p < 0.01, ### p < 0.001 as compared with exercise group.

Levels of muscle SREBP-1c, LXRs, ACLY, FAS (Fig. 5 Panel A-D) proteins were lower in the regularly exercised rats than the control rats (*P* < 0.001 for all). There were 18.5%, 24.6%, 23.3% and 23.7% decreases in SREBP-1c, LXRs, ACLY, FAS levels of muscle in response to exercise rats. In addition, muscle SREBP-1c, LXRs, ACLY, FAS levels in the regular exercise + CAPs group was significantly lower than that of the control and CAPs alone and regular exercise groups (*P* < 0.05; Fig. 5). SREBP-1c, LXRs, ACL, FAS levels decreased by 39.3%, 52.2%, 47.1%, and 52.4%, respectively (*P <* 0.05 for all) in exercised + CAPs rats compared with the control group. Peroxisome proliferator-activated receptors (PPAR-γ) level was higher in the regular exercise and CAPs alone than the control rats (Fig. 5 Panel E). There were 42.0% and 39.3% increases in PPAR-γ levels of muscle in the exercise and CAPs rats compared to control rats. PPAR-γ level increased by 29.4%, in exercised + CAPs rats compared with the control groups (*P <* 0.05). p-AMPK and total AMPK levels in the gastrocnemius muscle were reported in Fig. 5 (Panel F and G). Regularly exercised rats had greater total AMPK levels in muscle than control rats (*P* < 0.001). Regular exercise + CAPs treated rats had greater total AMPK and p-AMPK levels in muscle than regular exercise and control rats (*P* < 0.001).

There were positive correlations between triglyceride and lactate concentrations (r = 0.814), and SREBP-1c, (r = 0.838), LXRs, (r = 0.833), ACLY, (r = 0.843), FAS (r = 0.817) levels. However, there was a negative correlation between MDA and Nrf2 (r=-0.554) and HO-1 (r=-0.907) levels and positively correlated with NF-κB (r = 0.792) and IL-10 (r = 0.507) levels. Moreover, antioxidant enzyme activities were positively correlated with levels of Nrf2, HO-1, PPAR-γ, and p-AMPK, whereas was negatively correlated with levels of NF-κB, IL-10. Additionally, the Nrf2 level was negatively correlated with levels of IL-10 and HO-1 was positively correlated with p-AMPK.

## 4 Discussion

### 4.1 Effects of CAPs on the lipid metabolism, antioxidant status, and lactate levels

*Capsicum* has been shown to help improve lipid metabolism, endothelial function, hormone function, stabilize blood glucose and reduce oxidative stress [[35], [36], [37]]. Consistent with our results, there are several studies on the regular exercise lowering lipid parameters and increasing antioxidant status [5,7,38]. However, there are no reports on how lipid parameters and antioxidant status are altered by administration of CAPs to exercise-treated rats. For the first time in this study, we have shown the effect of co-administration of capsicum and regular exercise on these parameters. Nevertheless, there are some studies about the use of phytochemicals and antioxidants other than CAPs with regular exercise [7,8,19]. In a study, it was reported that exercise, however, caused a significant decrease in triglyceride, which was further reduced in Q10 supplemented rats [19]. In another study, plasma MDA levels were found to be lower in the chronic exercise group than controls, and it was lowered with antioxidant supplementation [39]. These effects of CAPs can be attributed to its role in energy consumption and fat metabolism. Furthermore, the effect of CAPs may be related to the effect of the PPAR-γ and TRPV-1 expression/activation [40].

In the present study, exercise, CAPs and their combination decreased serum lactate levels (Fig. 1 Panel D). This can be caused by exercise increasing blood flow efficiency and increasing the availability of mitochondrial oxygen for cellular respiration [41]. In addition, capsaicin increases metabolic rate and might cause glycolysis to be more efficient especially if the oxygen concentration is not rated limiting for oxidative phosphorylation [40]. It was also reported that lactate inhibits activation of the TRPV1 involved in energy metabolism by capsaicin [40].

### 4.2 Effects of CAPs on the muscle NF-κB and Nrf2 pathways

Regular exercise is known for enhancing the levels of anti-inflammatory and antioxidant markers such as NF-κB, IL-10, tumor necrosis factor-α (TNF-α) and Nrf2 [12,19,42]. The NF-κB pathway plays an important role in the induction of oxidative stress. Apart from reactive oxygen species, many inducers of the NF-κB pathway such as TNF-α, can give rise to oxidative stress, suggesting that the generation of reactive oxygen species (ROS) is not the only inducer of the NF-κB pathway [8]. Although the anti-inflammatory effect of regular exercise [12,19] and CAPs alone are well documented, no studies have been conducted on the effect of CAP administration with regular exercise. Consistent with previous studies, in the current study, we observed that regular exercise alone diminished inflammatory and antioxidant response by decreasing NF-κB and IL-10 levels. This study demonstrates that regular exercise and CAPs affected the ability of oxidative stress to activate NF-κB (p65), indicating that they play a key role in inhibition of the NF-κB pathway, which translocates the latent transcription factor to the nucleus. These markers are indicators for inflammation and related diseases and by increasing Nrf2 pathway and AMPK, a metabolic sensor and a key enzyme in muscle metabolism levels [8,42,43]. Tang et al. [44] reported that capsaicin inhibits lipopolysaccharide (LPS)-stimulated IL-1β, IL-6, TNF-α and NF-κB production in a time- and dose-dependent manner. In addition, capsaicin suppressed the migration and invasion of cholangiocarcinoma cells by inhibiting NF-κB via AMPK-SIRT1 and AMPK-IκBα signaling pathways, leading to the suppression of matrix metalloproteinase-9 (MMP-9) expression [45]. Nrf2 is a transcription factor that binds to the antioxidant response element (ARE), thereby increasing the transcription of various cytoprotective genes [[46], [47], [48]] and play a role in the induction of phase II detoxifying/antioxidant defense mechanisms to cope with oxidative stress through enhancing the expression of a number of enzymes, such as NAD(P)H quinone oxidoreductase 1, glutamate-cysteine ligase, HO-1, glutathione S-transferase and UDP-glucuronosyltransferase [47]. This study demonstrates an increase in Nrf2 with a concomitant increase in HO-1 in CAPs and exercise + CAPs groups. Uruno et al. [49] demonstrated that Nrf2 regulates skeletal glycogen metabolism, particularly glycogen-branching enzyme in the context of a maximal incremental treadmill protocol.

### 4.3 Effects of CAPs on the muscle PPAR-γ and AMPK levels

PPAR-γ may play a role in the regulation of adipocyte differentiation [50], lipid metabolism [51] and expression of target gene in glucose homeostasis [52]. In previous studies, it was shown that exercise increased the level of PPAR-γ in muscle [53,54]. In the present study, we demonstrated that combination of regular exercise and CAPs significantly increased the expression of muscle PPAR-γ. There are no earlier studies associated with examining the effects of CAPs treatment on the expression of muscle PPAR-γ in regularly exercised rats to compare with this study.

Mitochondrial biogenesis is an important process for cell viability and survival because dysfunction prevents the degradation of energy production and metabolism regulation as well as oxidative stress resistance [55,56]. Mitochondrial biogenesis is induced by activation of the AMPK pathway and by the expression of peroxisomal proliferator activator receptor c coactivator la (PGC-la), nuclear respiratory factors (NRF1 and NRF2) and mitochondrial transcription factor A (Tfam) [[55], [56], [57], [58]]. AMPK also regulates energy metabolism by increasing the gene expression of PGC-1α and SIRT1, which is involved in fatty acid oxidation [57,59]. In the present study, we observed that regular exercise combined with CAPs increased muscle pAMPK and total AMPK (Fig. 5, Panel H and G). Similar to our results, Colombo and Moncada [60] reported that the AMPK cascade participates in the cellular response to ROS-induced oxidative stress damage via increased expression levels of Nrf2, MnSOD, and catalase (CAT).

### 4.4 Effects of CAPs on the muscle SREBP-1c, LXRs, ACLY, and FAS levels

Several studies have shown that SREBP-1c induces mRNAs encoding enzymes catalyzing numerous steps in fatty acid and TG synthesis pathways, such as ACLY, acetyl-CoA synthetase (ACS), ACC, FAS, and stearoyl-CoA desaturase-1 (SCD1) [16,61,62]. In the present study, we demonstrated for the first time that combination of regular exercise and CAPs intake significantly reduced the levels of muscle SREBP-1c, LXRs, ACLY, and FAS transcriptional regulators that induce key lipogenic enzymes to promote lipogenesis. Similarly, previous studies have shown that physical exercise has a better lipidic profile compared to sedentary ones such as higher HDL-c and lower LDL-c and VLDL-c, and capsaicinoid consumption also positively affects the expression of lipogenic genes in adipose tissue [34,38,63]. Nevertheless, it has been reported that liver mRNA expression of FAS decreased and the expression levels of hormone-sensitive triglyceride lipase increased by capsaicin and paprika seed oil in rats [64].

In conclusion, this study shows that the combination of regular exercise and CAP stimulation increases the exercise capacity resulting in decreased inflammation and oxidative stress by suppressing muscle NF-κB and increasing Nrf2 / HO-1 pathways. Moreover, we demonstrate that they may inhibit the synthesis of fatty acid and promote fat oxidation in the healthy rat by downregulation of the muscle SREBP-1c, LXRs, ACLY, FAS levels and upregulation of muscle PPAR-γ and pAMPK levels.

## Conflicts of interest

The authors declare no conflict of interest. Vijaya Juturu is employed by OmniActive Health Technologies, Inc. in Morristown, NJ, USA.

## Transparency document

Transparency document
